# Outcomes after antiretroviral therapy during the expansion of HIV services in Haiti

**DOI:** 10.1371/journal.pone.0175521

**Published:** 2017-04-24

**Authors:** Margaret L. McNairy, Patrice Joseph, Michelle Unterbrink, Stanislas Galbaud, Jean-Edouard Mathon, Vanessa Rivera, Deanna Jannat-Khah, Lindsey Reif, Serena P. Koenig, Jean Wysler Domercant, Warren Johnson, Daniel W. Fitzgerald, Jean W. Pape

**Affiliations:** 1 Center for Global Health, Weill Cornell Medical College, New York, New York, United States of America; 2 Division of General Medicine, Weill Cornell Medical College, New York, New York, United States of America; 3 Haitian Study Group for Kaposi's Sarcoma and Opportunistic Infections (GHESKIO), Port-au-Prince, Haiti; 4 Brigham and Women’s Hospital, Harvard Medical School, Boston, Massachusetts, United States of America; 5 Centers for Disease Control and Prevention, Port-au-Prince, Haiti; Vanderbilt University, UNITED STATES

## Abstract

**Background:**

We report patient outcomes after antiretroviral therapy (ART) initiation in a network of HIV facilities in Haiti, including temporal trends and differences across clinics, during the expansion of HIV services in the country.

**Methods:**

We assessed outcomes at 12 months after ART initiation (baseline) using routinely collected data on adults (≥15 years) in 11 HIV facilities from July 2007-December 2013. Outcomes include death (ascertained from medical records), lost to follow-up (LTF) defined as no visit > 365 days from ART initiation, and retention defined as being alive and attending care ≥ 365 days from ART initiation. Outcomes were compared across calendar year of ART initiation and across facilities. Risk factors for death and LTF were assessed using Cox proportional hazards and competing risk regression models.

**Results:**

Cumulatively, 9,718 adults initiated ART with median age 37 years (IQR 30–46). Median CD4 count was 254 cells/uL (IQR 139–350). Twelve months after ART initiation, 4.4% (95% CI 4.0–4.8) of patients died, 21.7% (95% CI 20.9–22.6) were LTF, and 73.9% (95% CI 73.0–74.8) were retained in care. Twelve-month mortality decreased from 13.8% among adults who started ART in 2007 to 4.4% in 2013 (p<0.001). Twelve-month LTF after ART start was 29.2% in 2007, 18.7% in 2008, and increased to 30.1% in 2013 (p<0.001). Overall, twelve-month retention after ART start did not change over time but varied widely across facilities from 61.1% to 86.5%.

**Conclusion:**

Expansion of HIV services across Haiti has been successful with increasing numbers of patients initiating ART and decreasing twelve-month mortality rates. However, overall retention has not improved, despite differences across facilities, suggesting additional strategies to improve engagement in care are needed.

## Introduction

Over the past decade, the scale-up of HIV services, particularly access to antiretroviral therapy (ART), in resource-poor settings has been enormously successful with over 18 million adults initiating treatment [1]. While these efforts have saved millions of lives, many patients fail to realize the benefits of ART due to discontinuation of treatment, loss to follow-up (LTF), or death within the first year of treatment [2–9]. Though the Caribbean is the region most heavily affected by HIV outside of sub-Saharan Africa, published data on HIV outcomes from the region are limited [7, 9–12]. Moreover, the available data from HIV programs in the Caribbean have focused on health outcomes during the earliest era of HIV scale up, when HIV services were primarily delivered in specialty clinics and hospitals prior to the expansion and decentralization of HIV services across the country [9, 13–15].

Haiti is the poorest country in the Western Hemisphere with the highest adult HIV prevalence in the region, which was 6.2% in 1993 and decreased to 1.7% in 2014 [16–19]. An estimated 140,000 adults and 10,000 adolescents currently live with HIV in Haiti, and two-thirds of cases are in Port-au-Prince [20]. The Haitian Study Group for Kaposi's Sarcoma and Opportunistic Infections (GHESKIO) is a non-governmental organization that was established in 1982 as the first HIV/AIDS clinic in Latin America and the Caribbean [21]. In 2003, GHESKIO obtained international funding to provide free ART to eligible patients at its HIV specialty clinic in Port-au-Prince and has demonstrated dramatic improvements in HIV survival with the provision of ART [13, 22, 23]. In 2005, GHESKIO partnered with the Haitian Ministry of Public Health and Population (MSPP) with support from the U.S. President’s Emergency Plan for AIDS Relief (PEPFAR) to expand HIV testing, care, and treatment across all regions in Haiti in an effort to further decentralize HIV services to non-HIV specialty centers within Port-au-Prince and to facilities in rural areas. Through this effort, GHESKIO began supporting clinics across the country, referred to as the GHESKIO-MSPP network.

The objective of this analysis is to evaluate outcomes during the first twelve months after ART initiation among adults starting treatment within the GHESKIO-MSPP network from 2007 through 2013. We evaluate mortality, LTF and retention rates at 12-months after ART initiation, as well as temporal trends and differences across clinics.

## Methods

### Study setting and population

The study population includes adults ≥ 15 years who initiated ART in the GHESKIO-MSPP network of HIV facilities with electronic medical records (EMR) from July 1, 2007 through December 31, 2013. While a small number of network facilities provided ART as early as 2005, patient-level data was not routinely collected in the iSanté EMR system supported by the MSPP until in July 2007 [24, 25]. Among 22 facilities in the GHESKIO-MSPP network, 11 had an available iSanté EMR during the study period. One facility (clinic 11) only had available data in the iSanté database through December 31, 2012. All patients had a minimum study follow-up time of 12 months from time of ART initiation. Longitudinal data on pre-ART patients was not routinely collected in the EMR.

### HIV services

Eligibility for ART initiation followed national and WHO guidelines and included WHO Stage IV and/or CD4 count ≤ 200 cells/uL from 2007 to 2009, and WHO stage III or IV and/or CD4 count ≤ 350 cells/uL from 2009 through 2013 [26, 27]. From 2007 to 2009, the first-line ART regimen was zidovudine, lamivudine, and efavirenz or nevirapine, with single-drug substitutions permitted as outlined by WHO. Tenofovir-based regimens became first-line treatment in 2010. Clinic visits were monthly for the first three months and then once every three months. Medications were dispensed monthly to patients. CD4 counts were performed at time of ART initiation and every six months thereafter; viral load was not routinely measured. Retention in care and adherence to ART were encouraged by peer counseling and social support programs. Field workers attempted to find patients who missed a clinic appointment for up to six months from the date of the missed appointment to ascertain vital status.

### Clinical and facility measures

Sociodemographic and clinical measurements at the time of ART initiation included sex, age, HIV testing point of entry, body weight (kilograms), height (centimeters), WHO Stage, CD4 count, tuberculosis (TB), and ART regimen. Body mass index (BMI) was calculated as kilogram/meter^2^. Date of ART initiation was defined as the first date that ART was dispensed during the study period, with no prior history of documented ART. WHO stage, CD4 count, weight and height measurement at time of ART were defined as the measurement closest to ART start date within a 3-month window period. A diagnosis of TB was defined as a documented TB diagnosis in the iSanté database at the date of ART initiation. Guidelines for TB diagnosis in Haiti include presence of TB symptoms, chest x-ray suggestive of TB, and positive microscopy and or culture which is consistent with other studies [23, 28]. Clinic visit and pharmacy pick-up dates were included from date of ART initiation through date of study censor (October 1, 2015).

Patient information was documented by health care providers on national patient forms and subsequently entered by trained data clerks into the iSanté EMR. Data quality assessments were done quarterly to check for completeness and accuracy comparing paper records to data in the electronic database.

Facility-level characteristics included facility type (primary, secondary or tertiary) and facility location (urban or rural/semi-urban) based on characteristics provided by MSPP, as well as clinic size (number of patients cumulative on ART during the study period).

### Definitions of outcomes

Patient outcomes are mutually exclusive and included death, lost to follow-up (LTF) and retention in care at 12 months after date of ART initiation. Death refers to all-cause mortality documented in the EMR within 365 days of ART initiation. Lost to follow-up at twelve months after ART initiation was defined as no documented death or transfer prior to 365 days and no clinic visit after 365 days from date of ART initiation. Retention at twelve months was defined as being alive and attending clinic with documented visits in the medical record greater or equal to 365 days from the date of ART initiation.

### Statistical analysis

De-identified data on sociodemographic and clinical variables were extracted from the EMR. The probability of mortality after ART initiation was estimated using Kaplan Meier methods, censoring patients who were lost to follow-up at the time of their last visit. The cumulative incidence of LTF after ART initiation was estimated by treating death as a competing risk as outlined by Coviello and Boggess [29]. Patients who transferred were censored at the date of transfer. The Cochran-Armitage test was used to assess for trends in mortality, LTF and retention at 12 months after ART initiation over time from 2007 to 2013. Risk factors at ART initiation for mortality and LTF were assessed using Cox proportional hazard models, accounting for within-clinic correlation between patients with robust sandwich error terms, and sub-distribution hazard ratios (methods of Fine & Gray), respectively [30]. Missing data were imputed using the following predictors: sex, age, BMI, CD4 count, WHO stage, documented TB, year of ART initiation, clinic location and clinic size. We used STATA statistical software (Version 13.0, College Station, Tx) for all analyses.

### Ethics

This analysis was approved by the institutional review boards at Weill Cornell Medicine, Centers for Disease Control, and the Ethics Board at GHESKIO.

## Results

### Patient and facility characteristics

A total of 9,718 adults ≥ 15 years initiated ART in 11 GHESKIO-MSPP HIV facilities across Haiti (Table 1). The majority (63%) of patients were female and median age was 37 years (IQR 30–46). Only 855 (9%) were adolescents and youth ages 15–24 years. The majority of patients were tested for HIV at a voluntary counseling and testing site, with less than 10% tested by provider-initiated counseling and testing. Median CD4 count was 254 cells/uL (IQR 139–350), with 28% of patients missing a CD4 count. The proportion of patients with a missing CD4 count at time of ART initiation decreased from 36% in 2007 to 23% in 2013 (p< 0.001). One-quarter of patients had advanced WHO Stage III/IV disease at time of ART start, with 35% missing documentation of WHO Staging. Median weight at time of ART initiation for women was 55 kg (IQR 48–63) and 59 kg (IQR 54–66) for men. Fifteen percent of patients were underweight per the WHO definition of BMI < 18.5 kg/m^2^ [31]. A total of 483 (5%) patients had documented TB at time of ART initiation.

**Table 1:** Patient and facility characteristics at time of antiretroviral therapy initiation in GHESKIO-supported HIV facilities in Haiti from July 2007-December 2013 (N = 9,718).

Characteristic	N (%)
Female	6145 (63.2%)
**Age**	
Median (IQR)	37 (30–46)
15–19	169 (1.7%)
20–24	686 (7.1%)
25–39	4716 (48.5%)
40–44	1370 (14.1%)
≥ 45	2679 (27.6%)
Missing	98 (1.0%)
**HIV testing point of entry**	
Voluntary counseling and testing (VCT)	3153 (32.4%)
Provider initiated counseling and testing (PICT)	876 (9.0%)
Community-based testing	748 (7.7%)
Prevention of Mother to Child Transmission program	267 (2.7%)
Missing	4674 (48.1%)
**WHO stage**	
Stage I	1599 (16.5%)
Stage II	2246 (23.1%)
Stage III	1517 (15.6%)
Stage IV	990 (10.2%)
Missing	3366 (34.6%)
**CD4 cell count (cells/uL)**	
Median (IQR)	254 (139–350)
< 100	1290 (13.3%)
100–199	1339 (13.8%)
200–349	2583 (26.6%)
> = 350	1809 (18.6%)
Missing	2697 (27.8%)
**Weight (kg)**	
Median all patients (IQR)	56.7 (49.9, 64.2)
Median men (IQR)	59.4 (53.5, 65.5)
Median women (IQR)	54.9 (48.0, 63.0)
Missing	1226 (12.6%)
**BMI (kg/m2)**	
< 18.5	1437 (14.8%)
18.5–24.9	4881 (50.2%)
25–29.9	1002 (10.3%)
≥ 30	284 (2.9%)
Missing	2114 (21.8%)
**Documented tuberculosis**	483 (5.0%)
**Antiretroviral Regimen**	
zidovudine, lamivudine, efavirenz/nevirapine	6840 (70.4%)
tenofovir, lamivudine/efavirenz/nevirapine	2027 (20.9%)
Other	851 (8.8%)
**Year of Antiretroviral Initiation**	
2007	264 (2.7%)
2008	1058 (10.9%)
2009	1373 (14.1%)
2010	1429 (14.7%)
2011	1989 (20.5%)
2012	1841 (18.9%)
2013	1764 (18.2%)
**Facility type**	
Secondary/tertiary	5985 (61.6%)
Primary	3733 (38.4%)
**Facility location**	
Urban	4994 (51.4%)
Rural/semi-urban	4724 (48.6%)
**Facility Size**	
Median (IQR)	1514 (535–1842)
Range	437–2125
< 1000 patients	4237 (43.6%)
> 1000 patients	5481 (56.4%)

**Fig 1:**
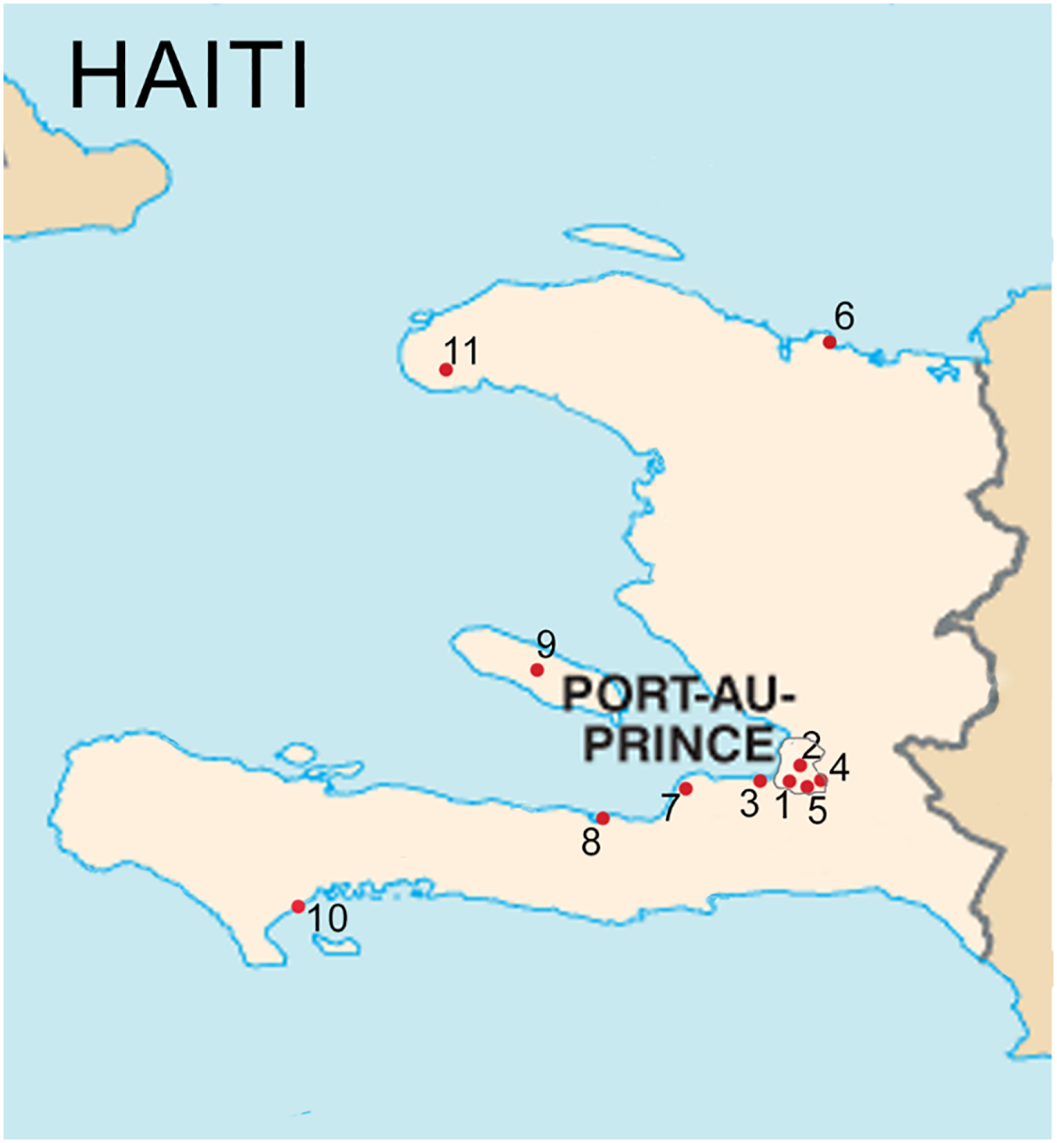
Map of included HIV facilities (N = 11) in Haiti.

Fig 1 details the location of the 11 GHESKIO-MSPP network facilities. Five facilities were located in Port-au-Prince and the remaining six in other provinces. The median number of patients initiated on ART per facility was 1,514 (IQR 535–1,842, range 437–2,125) during the study period. The number of patients who initiated ART increased annually from 264 (3% of all patients in the analysis) in 2007 to 1,989 (21%) in 2011, and subsequently plateaued in 2012 and 2013 (Table 1). Half of patients initiated ART in urban HIV facilities and 62% in secondary/tertiary facilities.

### Patient outcomes

Twelve-month outcomes after ART initiation among all patients who initiated ART from 2007 through 2013 include the following: 4.4% (95% CI 4.0–4.8) patients died, 21.7% (95% CI 20.9–22.6) were LTF, and 73.9% (95% CI 73.0–74.8) were retained in care.

#### Patient characteristics and outcomes after ART initiation over time

From 2007 to 2013, the median age at time of ART initiation remained greater than 35 years and the majority of patients were female (Table 2). Among 7,021 (72%) patients with a CD4 count at time of ART initiation, the median CD4 count increased from 152 cells/uL (IQR 73–237) in 2007 to 293 cells/uL (IQR 171–418) in 2013 (p<0.001). Among 6,352 (65%) patients with a documented WHO Stage, the proportion with Stage III/IV decreased from 59% to 38% over time (p<0.001).

**Table 2:** Patient characteristics and outcomes of adults initiating antiretroviral therapy by calendar year of initiation and by HIV facility.

	No. of Patients	Median follow-up time days (IQR)	Median ART pickups (IQR)	Age Median(IQR)	Female N (%)	CD4 CountMedian(IQR)	WHO Stage III/IVN (%)	12 month Mortality (95% CI)	12 month Lost to Follow-Up (95% CI)	12 month Retention (95% CI)
**Year of ART Initiation**										
**2007**	264	N/A	N/A	38 (32–46)	160 (60.1)	152 (73–237)	107 (59.4)	13.8 (9.8–19.1)	28.0 (22.7–33.5)	60.2 (54.1–65.8)
**2008**	1058	N/A	N/A	38 (31–45)	662 (62.6)	190 (95–292)	332 (48.6)	5.5 (4.3–7.2)	18.3 (16.0–20.7)	76.6 (73.9–79.1)
**2009**	1373	N/A	N/A	37 (30–46)	796 (58.0)	219 (131–299)	423 (44.2)	5.2 (4.1–6.6)	17.8 (15.8–19.8)	77.5 (75.2–79.6)
**2010**	1429	N/A	N/A	38 (30–46)	908 (63.5)	226 (117–321)	398 (40.7)	5.4 (4.3–6.7)	15.3 (13.4–17.2)	79.7 (77.5–81.7)
**2011**	1989	N/A	N/A	37 (30–45)	1268 (63.8)	275 (153–372)	472 (35.9)	3.5 (2.8–4.5)	20.7 (19.0–22.5)	76.2 (74.2–78.0)
**2012**	1841	N/A	N/A	37 (30–46)	1195 (64.9)	292 (168–437)	423 (32.3)	4.3 (3.4–5.4)	24.3 (22.3–26.3)	71.9 (69.8–73.9)
**2013**	1764	N/A	N/A	36 (29–46)	1156 (65.5)	293 (171–418)	352 (37.9)	4.4 (3.5–5.6)	29.6 (27.5–31.7)	66.7 (64.5–68.9)
				p = 0.048	p<0.001	p<0.001	p<0.001	p<0.001	p<0.001	p = 0.081
**HIV Facility**										
**Clinic 1**	1514	805 (308–1393)	16 (7–30)	36 (30–44)	964 (63.7)	256 (153–340)	272 (25.7)	3.0 (2.2–4.1)	16.0 (14.1–17.8)	81.3 (79.2–83.2)
**Clinic 2**	1842	392 (15–932)	6 (2–13)	37 (30–45)	1265 (68.7)	308 (192–446)	793 (59.7)	1.7 (1.2–2.5)	37.5 (35.2–40.0)	61.1 (58.9–63.3)
**Clinic 3**	535	619 (245–1361)	11 (4–24)	38 (31–46)	361 (67.5)	168 (134–175)	60 (20.1)	2.4 (1.3–4.2)	23.2 (20.0–26.8)	74.8 (70.9–78.2)
**Clinic 4**	585	618 (150–1164)	11 (4–21)	38 (30–45)	378 (64.6)	358 (219–526)	106 (22.6)	7.0 (5.1–9.4)	18.8 (15.8–22.1)	74.5 (70.8–77.9)
**Clinic 5**	518	623 (250–1058)	15 (7–27)	35 (29–43)	330 (63.7)	285 (204–358)	33 (12.6)	1.6 (0.8–3.3)	25.3 (21.6–29.1)	73.6 (69.5–77.1)
**Clinic 6**	475	711 (224–1326)	13 (6–23)	37 (31–45)	288 (60.6)	237 (124–342)	148 (49.7)	5.9 (4.0–8.5)	13.9 (11.0–17.2)	80.8 (77.0–84.1)
**Clinic 7**	452	560 (124–1038)	6 (3–10)	42 (34–50)	226 (50.0)	168 (64–307)	103 (47.5)	6.2 (4.2–9.1)	31.4 (27.2–35.7)	63.7 (59.1–68.0)
**Clinic 8**	719	667 (296–1232)	16 (8–29)	36 (29–45)	439 (61.1)	270 (124–387)	139 (30.3)	6.4 (4.8–8.6)	13.2 (10.9–15.8)	80.7 (77.6–83.4)
**Clinic 9**	437	861 (463–1556)	18 (10–33)	37 (30–47)	283 (64.8)	199 (73–314)	182 (63.0)	7.8 (5.6–10.8)	5.9 (4.0–8.4)	86.5 (82.9–89.4)
**Clinic 10**	2125	610 (161–1249)	10 (5–18)	37 (30–47)	1193 (56.1)	219 (102–314)	562 (39.9)	6.5 (5.5–7.7)	20.4 (18.7–22.1)	73.6 (71.7–75.4)
**Clinic 11**	516	786 (440–1255)	17 (10–27)	37 (30–46)	331 (64.1)	234 (153–314)	109 (41.0)	8.7 (6.5–11.6)	10.2 (7.8–13.1)	81.6 (78.0–84.7)
				p<0.001	p<0.001	p<0.001	p<0.001	p<0.001	p<0.001	p<0.001

P values: Cochrane-Armitage test use for trend in 12-month outcomes. Chi Square for differences in sex and WHO Stage; log-rank test for differences in 12-month outcomes across clinics; Kruskal-Wallis for differences in age and CD4 count.

**Fig 2:**
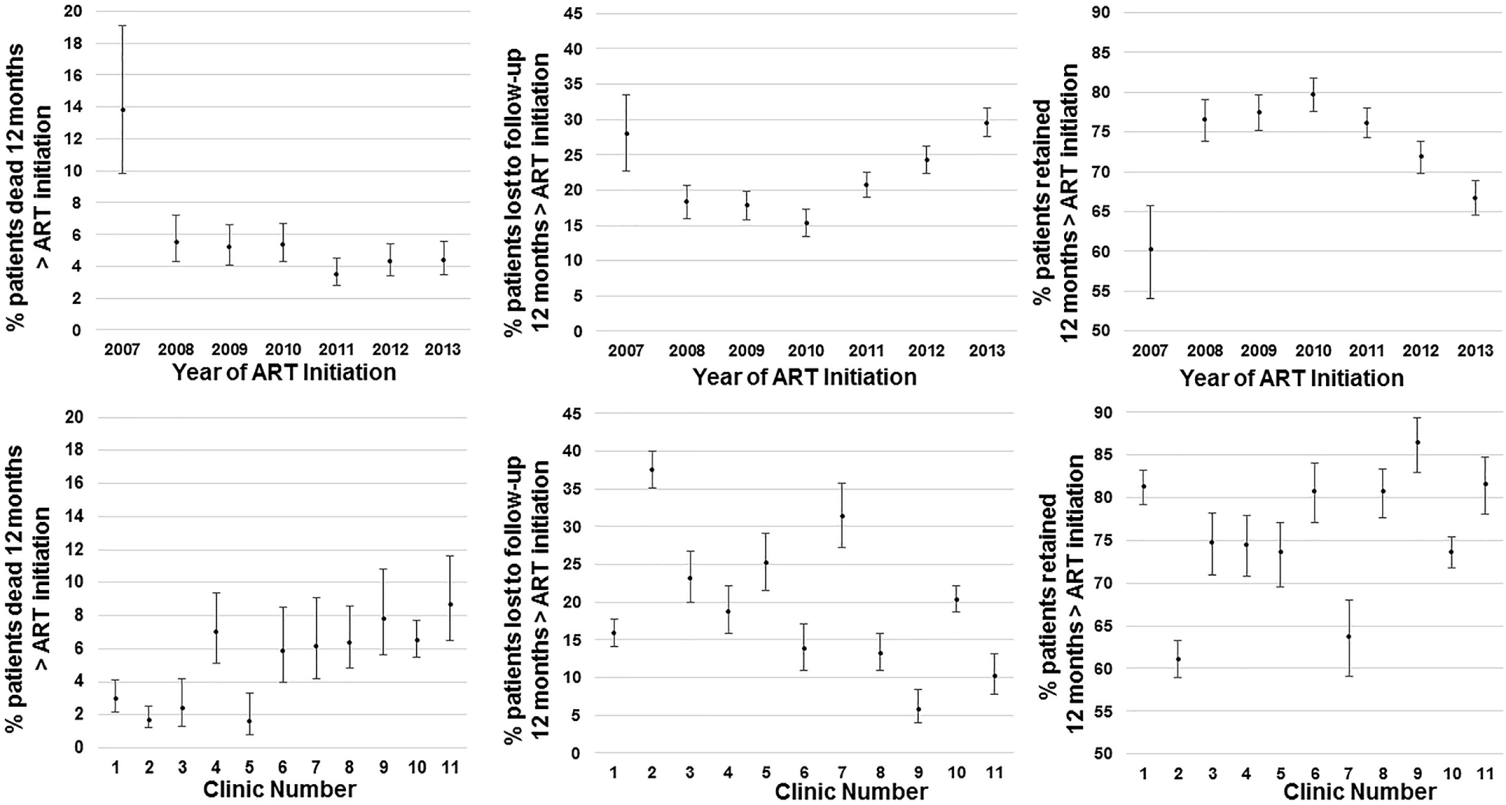
Death, lost to follow-up and retention at 12-months after ART initiation among adults initiating ART in GHESKIO-MSPP facilities in Haiti from 2007 to 2013. Median point estimates are plotted with 95% Confidence Interval bars.

The trend in the 12-month mortality rate after ART initiation decreased from 13.8% (95% CI 9.8–19.1) in 2007 to 4.4% (95% CI 3.5–5.6) in 2013 (p< 0.001) (Table 2, Fig 2). LTF rates at 12 months after ART initiation initially decreased from 28.0% (95% CI 22.7–33.5) in 2007 to 15.3% (95% CI 13.4–17.2) in 2010 and then increased to 29.6% (95% CI 27.5–31.7) in 2013, with an overall increasing trend from 2007 to 2013 (p< 0.001). Excluding data from 2007, a decreasing trend in mortality and increasing trend in LTF were still observed (p<0.001). There was no significant trend in retention at 12 months after ART initiation over time: 60.2% (95% CI 54.1–65.8) in 2007 as compared to 66.7% (95% CI 64.5–68.9) in 2013 (p = 0.081).

The overall mortality rate in the first three months was 7.1 deaths/100 person years as compared to 2.6 deaths/100 PY from one to five years after ART initiation among patients with follow-up time greater than one year (S1 Table). The mortality rate in the first year decreased from 17.8 deaths/100 PY in 2007 to 4.9 deaths/100 PY in 2013. The overall LTF rate in the first year after ART initiation was 26.7/100 PY as compared to 14.4/100 PY after the first year through year five. Approximately one-third of patients who were lost in the first year only attended one clinic visit (N = 625/2106, 30%). The proportion of one-visit-only patients decreased three fold from 2007 to 2013 (S1 Table).

#### Differences across HIV facilities

Across HIV facilities, median age differed from 35 years to 42 years (p< 0.001) (Table 2). Median CD4 count at time of ART initiation ranged widely from 168 cells/ul to 358 cells/ul across facilities (p<0.001). The proportion of patients with WHO Stage III/IV varied from 13% to 63% (p<0.001). The clinics with the highest proportion of patients with advanced WHO Stage were facilities in Port-au-Prince and those that provided treatment for both HIV and TB.

Mortality rates at 12-months after ART initiation ranged from 1.6% (95% CI 0.8–3.3) to 8.7% (95% CI 6.5–11.6) across clinics (p< 0.001) (Table 2, Fig 2). Rates of LTF at 12-months after ART initiation ranged even more dramatically from 5.9% (95% CI 4.0–8.4) to 37.5% (95% CI 35.2–40.0) (p< 0.001). Overall retention at 12-months ranged from 61.1% (95% CI 58.9–63.3) to 86.5% (95% CI 82.9–89.4) (p< 0.001).

#### Predictors of death and lost to follow-up at 12-months after ART initiation

Individual-levels factors associated with death at 12-months after ART initiation include male gender (adjusted hazard ratio (aHR) 1.3, 95% CI 1.1–15), WHO Stage III/IV (aHR 1.3, 95% CI 1.0–1.7), CD4 count < 100 cells/uL (aHR 2.6, 95% CI 1.7–4) and BMI < 18.5 kg/m2 (aHR 2.0, 95% CI 1.3–3.1) (Table 3). No facility-level factor was associated with increased risk of death. There was no interaction between CD4 count cells/uL and calendar year.

**Table 3:** Patient and facility characteristics associated with death and lost to follow-up at 12 months after antiretroviral initiation.

	Death12 months after ART Initiation				Lost to Follow-Up 12 months after ART Initiation			
	UnadjustedHR	95% CI	AdjustedHR	95% CI	UnadjustedSHR	95% CI	AdjustedSHR	95% CI
**Patient Characteristic**								
**Sex**								
Female	reference							
Male	1.5*	1.3–1.8	1.3*	1.1–1.5	1.0	0.9–1.1	1.1	1.0–1.2
**Age category**								
15–19	1.2	0.6–2.4	1.0	0.5–2.1	1.1	0.8–1.5	1.0	0.7–1.3
20–24	Reference							
25–39	1.0	0.7–1.4	0.9	0.6–1.4	0.7*	0.6–0.8	0.6*	0.6–0.7
40–44	1.2	0.9–1.7	1.0	0.7–1.4	0.6*	0.5–0.7	0.5*	0.4–0.6
> = 45	1.4	0.9–2.0	1.1	0.7–1.6	0.5*	0.4–0.6	0.5*	0.4–0.6
**WHO Stage**								
I/II	Reference							
III/VI	1.7*	1.2–2.4	1.3^	1.0–1.7	1.4*	1.2–1.5	1.3*	1.2–1.5
**CD4 Count (cells/uL)**								
<100	4.2*	3.0–6.1	2.6*	1.7–4.0	1.0	0.9–1.2	1.1^	1.0–1.3
100–199	2.0*	1.3–3.1	1.5^	1.0–2.4	0.7*	0.6–0.8	0.8*	0.7–1.0
200–349	1.3	0.8–2.0	1.1	0.7–1.7	0.8*	0.7–0.9	0.8*	0.7–0.9
>350	reference							
**BMI (kg/m**^**2**^**)**								
< 18.5	2.9*	2.0–4.1	2.0*	1.3–3.1	1.0	0.9–1.2	1.1	0.9–1.2
≥18.5	Reference							
**Documented TB**								
No	Reference							
Yes	1.3	0.8–2.1	0.7	0.4–1.2	1.0	0.8–1.2	1.0	0.8–1.3
**Calendar year of initiation**								
2007	Reference							
2008	0.4*	0.2–0.7	0.5*	0.3–0.8	0.6*	0.4–0.8	0.6*	0.4–0.8
2009	0.4*	0.2–0.6	0.4*	0.3–0.7	0.6*	0.4–0.7	0.6*	0.5–0.8
2010	0.4*	0.2–0.7	0.5	0.3–1.0	0.5*	0.3–0.6	0.5*	0.4-.07
2011	0.2*	0.1–0.5	0.4*	0.2–0.8	0.7*	0.5–0.9	0.7*	0.5–0.9
2012	0.3*	0.2–0.5	0.4*	0.2–0.8	0.8^	0.6–1.0	0.9	0.7–1.1
2013	0.3*	0.1–0.6	0.5	0.2–1.1	0.9	0.7–1.2	1.0	0.8–1.3
**Facility Characteristics**								
**Location**								
Urban	Reference							
Rural	2.4*	1.5–3.9	1.7	0.9–3.1	0.7*	0.6–0.7	0.7*	0.7–0.8
**Facility Size**								
< 1000 patients	Reference							
≥1000 patients	0.7	0.3–1.4	0.8	0.5–1.3	1.8*	1.6–1.9	1.6*	1.5–1.8

*p value < 0.05,

^p value < 0.10

All patients older than or equal to 25 years, as compared to 20–24 year-olds, had a decreased risk of LTF. Patients with WHO Stage III/IV disease had a 30% increased risk of LTF as compared to patients with WHO Stage I/II disease (aSHR 1.3, 95% CI 1.2–1.5). Patients who initiated ART in HIV facilities with ≥ 1000 cumulative patients on ART during the study period, were at higher risk for LTF as compared to those as smaller clinics (aSHR 1.6, 95% CI 1.5–1.8). Additionally, patients who initiated ART at rural or semi-urban clinics, as compared to urban clinics, were less likely to be LTF (aSHR 0.7, 95% CI 0.7–0.8).

## Discussion

This cohort study of 11 HIV facilities includes approximately 10,000 patients and reports outcomes during the first year after ART initiation during a period of expansion of HIV services in Haiti. From 2007 through 2013, there was a steady increase in the number of patients initiating ART in this network of HIV facilities. At 12 months after ART initiation, 4.4% of the study population died, 21.7% were lost to follow-up, and 73.9% were alive and retained in care. While there was a decreased trend in mortality rates at 12-months after ART initiation, LTF increased such that there was ultimately no improvement in retention over time. Additionally, a substantial heterogeneity in outcomes was seen across clinics and warrants further investigation to understand best practices in clinics with optimal outcomes to share with poorer performing clinics.

This study’s overall mortality rate at 12 months after ART initiation of 4.4% is slightly lower than rates from other studies in low and middle-income countries and from patients treated at GHESKIO’s central HIV specialty clinic in Port-au-Prince [9]. In a meta-analysis of 25 HIV programs in Africa and Asia, 12-month mortality after ART initiation from 2003 to 2007 was 6.8% (10). In a meta-analysis of 7 countries in the Caribbean and Latin America, including patients from GHESKIO specialty clinic, from 2000–2013, one-year mortality after ART initiation was 5.4% [11]. Our cohort’s lower mortality could reflect higher CD4 counts at ART initiation, improvements in care, and under-documentation of deaths. For example, our cohort’s median CD4 count of 254 cells/uL is much higher than median CD4 counts of 100–120 cells/uL reported from cohorts in other low and middle-income countries including Haiti, and likely reflects changes in ART eligibility guidelines and earlier presentation for treatment [9, 14, 32].

Our finding of a decrease trend in 12-month mortality among patients initiating ART from 2007 to 2013 is similar to findings from an analysis of 12-month mortality trends across 8 clinics in South Africa, which reports mortality decreased from 8.9% in 2002 to 5.6% in 2007 [7]. Factors associated with one-year mortality in this study, including male gender, advanced WHO Stage and CD4 < 100 cells/uL, are similar to other studies [7, 10, 11]. Interestingly, even when controlling for all known factors, there is still an association between calendar year of ART initiation and mortality. Patients who initiated treatment in years after 2007, had a 50% lower risk of death as compared to those who initiated in 2007, which cannot be explained by other factors available in our dataset. This may be due to non-measurable factors such as improved clinician ART management with increased experience over time, fewer drug stock-outs, or improved diagnostic capacity and management of comorbid diseases and opportunistic infections.

Direct comparison of our results of LTF to other studies may be limited given differences in definitions of LTF across studies. Our rates of LTF are higher than the rate of 16.1 persons/100 PY who were lost in the first six months of treatment reported among patients who initiated ART between 1999 and 2008 in the GHESKIO HIV specialty clinic in Port-au-Prince [9]. Our results of an increasing trend in LTF over time are similar to LTF trends reported from other HIV programs in developing countries [7, 33–36]. Possible reasons for the increasing trend include increased undocumented transfers to other clinics, deteriorating retention in the face of increasing patient loads, and data management challenges [37–39]. Another reason could be increases in the number of unascertained deaths among patients who are categorized as lost; however, studies that have linked patient records to national vital registrations or have tracked lost patients to ascertain vital status have not found increasing rates of mortality over time [40, 41]. Larger program size was associated with increased risk of LTF in this analysis, which has been seen across other HIV programs in developing countries. In a meta-analysis of 25 HIV programs in Africa and Asia, program size was associated with a seven-fold increase in risk of LTF [10].

Undocumented, or silent, transfers are hypothesized to be a significant problem in Haiti by local HIV providers. Unfortunately, there is no unique national identification number in Haiti to track patients across clinics in the EMR. Our study found that patients who initiated ART in urban HIV facilities were more likely to be LTF which may be due, in part, to patients transferring without documentation to clinics closer to their homes in rural areas. Currently, the MSPP is evaluating a finger-print tracking system as a unique identifier of HIV patients across sites to better understand patient transfer and migration. The fact that 30% of patients who initiated ART in this cohort never returned to the clinic also points to a subgroup of LTF patients who may be distinct from patients who disengage later during care, and additional research is needed to understand each group’s unique barriers to care. Data management is another challenge given the poor physical infrastructure of the electronic medical record system in these clinics. In an assessment of data quality of variables in the iSanté database, TB status and ART eligibility were incomplete 20–40% of the time, and discontinuation forms, which document dates of transfer, death, and LTF status were entered late [42].

Of note, there are no significant differences in outcomes noted in the year following the historic earthquake which occurred in January 2010. Comparing outcomes among patients who initiated ART in the year prior to the earthquake in 2009 to 2010, the cumulative incidence of mortality was 5.2 (95% CI 4.1–6.6) versus 5.4 (95% CI 4.3–6.7) with overlapping confidence intervals. Lost to follow-up was also similar in 2009 at 17.8 (95%CI: 15.8–19.8) and in 2010 was 15.3 (95%CI: 13.4–17.2) with overlapping confidence intervals. This is a similar finding to previous analyses we have conducted pre- and post-earthquake reporting that the 2010 earthquake did not significantly impact outcomes of LTF or death among HIV patients [43].

Multiple interventions are being evaluated to improve retention in care among patients who start ART in Haiti. Early work with directly observed therapy for ART using community workers has shown promising results [44]. Fast tracking stable patients on ART, defined as patients who are retained in care at 6 months after ART start with excellent medication adherence, has also been associated with high retention in a pilot study at the GHESKIO HIV specialty clinic [45].

The marked heterogeneity in outcomes across facilities has been reported in other multi-country cohort studies [8, 9, 11, 36]. Differences could in part be explained by differences in undocumented deaths among patients categorized as lost. Other explanations include differences in demographics in the HIV epidemic across the country, internal migration and social and economic conditions across the country, as well as facility-level characteristics such as staffing, provider competency, laboratory and pharmacy equipment, and quality of care measures such as screening and treatment of opportunistic infections. Interestingly, the clinic with the highest 12-month retention (86.5%) and most balanced rates of death and lost to follow-up (7.8% and 6.2% respectively) was clinic 9 which is located on the small island of Ile de la Gonave where there may be lower migration rates to other clinics. In contrast, clinics 2, 3, and 4 had the poorest retention are located near slums of Port-au-Prince which are areas of high migration.

A strength of this study is the inclusion of a unique set of HIV facilities in Haiti that have not previously been reported. The majority of ART outcomes in Haiti have been previously reported from the GHESKIO central clinic in Port-au-Prince, which is a specialty clinic included in the CCASAnet cohort (Caribbean, Central and South American Network for HIV research), and a regional leader in both HIV research and service delivery [9, 11, 13, 21, 23, 28, 46]. Limitations of this analysis include the use of routinely collected patient data which lack vital status for patients who are categorized as lost and the inability to track patients who receive care at multiple facilities due to the lack of unique patient identifier. Additionally, we lack data on HIV transmission mode and risk behaviors to evaluate the scale up of services among vulnerable populations such as men who have sex with men and commercial sex workers. Despite these limitations, this cohort represents a large proportion of the clinics responsible for the rapid decentralization of ART across Haiti and is likely generalizable to other clinics with similar geographic and patient characteristics.

## Conclusion

Despite multiple social and economic challenges that occurred in Haiti during the study period, including political instability, a catastrophic earthquake in 2010, and a devastating cholera epidemic, Haiti was able to successfully expand HIV services across the country [15]. This study reports both the successes and ongoing challenges of the ongoing expansion of HIV services across Haiti and adds to the literature of ART outcomes among earlier cohorts of patients receiving care prior to 2007 in the Caribbean [9, 14]. While it is encouraging that mortality rates in the first year of treatment have steadily decreased over time, more work is needed to decrease high rates of loss to follow-up in order to optimize health outcomes and further curb the epidemic on a population level.

## Supporting information

S1 Table Rates of mortality and lost to follow-up after antiretroviral initiation and by HIV facility. (DOCX)
