# Supplementary material for: Outcomes after antiretroviral therapy during the expansion of HIV services in Haiti
Source: PLoS One. 2017 Apr 24;12(4):e0175521. doi: 10.1371/journal.pone.0175521 (PMC5402937; doi:10.1371/journal.pone.0175521)
Supplement: S1 Table — (DOCX) [file pone.0175521.s001.docx]

Supplement Table 2: Rates of mortality and lost to follow-up after antiretroviral initiation and by year of ART initiation and by HIV facility. Rates reported per 100 person years.

| **Mortality Rates by Year** | | | |
| --- | --- | --- | --- |
| **Year** | **Rate 0 to 1 Day (95% CI)** | **Rate 0 to 1 Year (95% CI)** | **Rate 1 to 5 years (95% CI)** |
| 2007 | 1807.8 (1070.7-3052.4) | 17.8 (12.5-25.4) | 3.8 (2.4-6.0) |
| 2008 | 763.1 (511.5-1138.6) | 6.3 (4.8-8.2) | 2.7 (2.1-3.4) |
| 2009 | 391.2 (239.7-638.5) | 5.7 (4.5-7.3) | 2.9 (2.4-3.5) |
| 2010 | 681.2 (473.4-980.3) | 6.0 (4.7-7.5) | 2.5 (2.0-3.1) |
| 2011 | 218.8 (127.0-376.8) | 3.8 (3.0-4.9) | 2.2 (1.7-2.8) |
| 2012 | 346.1 (220.8-542.6) | 4.8 (3.8-6.0) | 2.5 (1.8-3.4) |
| 2013 | 342.5 (215.8-543.6) | 4.9 (3.8-6.2) | 3.3 (2.1-5.2) |
| TOTAL | 459.3 (387.5-544.4) | 5.4 (4.9-5.9) | 2.6 (2.4-2.9) |
| **Lost to follow-up Rates by Year** | | | |
|  | **Rate 0 to 1 Day (95% CI)** | **Rate 0 to 1 Year (95% CI)** | **Rate 1 to 5 years (95% CI)** |
| 2007 | 6843.7 (5228.4-8958.0) | 42.6 (33.9-53.5) | 9.1 (6.8-12.2) |
| 2008 | 2734.6 (2213.6-3378.1) | 22.4 (19.5-25.8) | 8.3 (7.2-9.4) |
| 2009 | 2298.2 (1877.6-2813.1) | 21.4 (18.9-24.3) | 9.2 (8.3-10.3) |
| 2010 | 1996.7 (1614.3-2469.6) | 18.1 (15.8-20.6) | 15.0 (13.7-16.4) |
| 2011 | 1363.1 (1096.3-1694.7) | 24.8 (22.5-27.3) | 17.5 (16.1-19.1) |
| 2012 | 1949.0 (1612.6-2355.6) | 30.1 (27.4-33.0) | 27.0 (24.6-29.6) |
| 2013 | 2264.0 (1891.7-2709.7) | 38.0 (34.8-41.4) | 22.7 (19.1-27.0) |
| TOTAL | 2158.5 (1995.8-2334.6) | 26.7 (25.6-27.8) | 14.4 (13.8-15) |
| **Mortality Rates by HIV Facility** | | | |
| **HIV Facility** | **Rate 0 to 1 Day (95% CI)** | **Rate 0 to 1 Year (95% CI)** | **Rate 1 to 5 years (95% CI)** |
| 1 | 176.8 (88.4-353.5) | 3.2 (2.4-4.3) | 1.9 (1.4-2.4) |
| 2 | 202.2 (112.0-365.1) | 2.0 (1.4-3.0) | 1.6 (1.1-2.2) |
| 3 | 0.0 (.-.) | 2.5 (1.4-4.5) | 0.8 (0.4-1.7) |
| 4 | 1551.2 (1063.8-2262.0) | 8.2 (6.0-11.2) | 2.1 (1.3-3.4) |
| 5 | 0.0 (.-.) | 1.6 (0.8-3.4) | 1.1 (0.5-2.2) |
| 6 | 352.2 (146.6-846.1) | 6.4 (4.4-9.4) | 3.6 (2.5-5.1) |
| 7 | 669.4 (348.3-1286.6) | 7.2 (4.8-10.8) | 1.6 (0.8-3.1) |
| 8 | 605.0 (351.3-1042.0) | 7.1 (5.3-9.5) | 3.8 (2.8-5.1) |
| 9 | 458.8 (206.1-1021.2) | 8.3 (5.9-11.6) | 8.5 (6.8-10.7) |
| 10 | 661.9 (489.2-895.6) | 7.4 (6.2-8.8) | 2.0 (1.6-2.5) |
| 11 | 778.6 (442.2-1371.0) | 9.5 (7.0-12.8) | 6.4 (4.9-8.5) |
| **Lost to follow-up Rates by HIV Facility** | | | |
| **HIV Facility** | **Rate 0 to 1 Day (95% CI)** | **Rate 0 to 1 Year (95% CI)** | **Rate 1 to 5 years (95% CI)** |
| 1 | 1171.1 (894.7-1532.9) | 18.3 (16.2-20.8) | 7.6 (6.7-8.7) |
| 2 | 5771.2 (5166.9-6446.2) | 53.6 (49.7-57.7) | 17.4 (15.7-19.3) |
| 3 | 2824.5 (2108.9-3783.0) | 27.9 (23.4-33.3) | 17.6 (15.1-20.6) |
| 4 | 1608.7 (1110.7-2329.8) | 23.2 (19.2-27.9) | 11.5 (9.4-14.0) |
| 5 | 1615.6 (1091.7-2390.9) | 30.2 (25.4-35.9) | 30.9 (26.9-35.5) |
| 6 | 1056.5 (636.9-1752.5) | 16.0 (12.6-20.5) | 7.7 (6.0-9.8) |
| 7 | 2603.3 (1869.1-3625.7) | 42.1 (35.6-49.6) | 41.6 (36.4-47.6) |
| 8 | 884.3 (564.0-1386.4) | 15.2 (12.5-18.6) | 8.5 (7.0-10.4) |
| 9 | 458.8 (206.1-1021.2) | 6.5 (4.4-9.6) | 2.6 (1.7-3.9) |
| 10 | 1118.9 (886.7-1412.0) | 25.1 (22.8-27.6) | 11.0 (9.9-12.1) |
| 11 | 908.4 (538.0-1533.8) | 11.5 (8.7-15.1) | 41.3 (37.1-46.1) |
